# Association Between Blood Biochemical Factors Contributing to Cognitive Decline and B Vitamins in Patients With Alzheimer's Disease

**DOI:** 10.3389/fnut.2022.823573

**Published:** 2022-02-21

**Authors:** Ting Qian, Lei Zhao, Xiaoli Pan, Shaoming Sang, Yangqi Xu, Changpeng Wang, Chunjiu Zhong, Guoqiang Fei, Xiaoqin Cheng

**Affiliations:** ^1^Department of Neurology, Zhongshan Hospital (Xiamen Branch), Fudan University, Shanghai, China; ^2^State Key Laboratory of Medical Neurobiology, Institutes of Brain Science and Collaborative Innovation Center for Brain Science, Fudan University, Shanghai, China; ^3^Department of Neurology, Ninth People's Hospital, Shanghai Jiao Tong University, Shanghai, China

**Keywords:** malnutrition, cognitive dysfunction, Alzheimer's disease, B vitamins, TDP

## Abstract

**Background:**

Malnutrition, metabolism stress, inflammation, peripheral organs dysfunction, and B vitamins deficiency significantly contribute to the progression and mortality of Alzheimer's disease (AD). However, it is unclear which blood biochemical indicators are most closely related to cognitive decline and B vitamins deficiency (thiamine, folate, vitamin B12) in patients with AD.

**Methods:**

This was a cross-sectional study of 206 AD patients recruited from six hospitals in China. Thiamine diphosphate (TDP), the bioactive form of thiamine, was measured by high-performance liquid chromatography fluoroscopy (HPLC) at a single center. Levels of biochemical indicators (except TDP) were measured by regular and standard laboratory tests in each hospital. Pearson's rank correlation analysis was used to assess relationships between B vitamins and biochemical indicators. *T*-test was used to compare the difference between ApoE ε4 and non-ApoE ε4 groups. Differences were considered statistically significant as *P* < 0.05.

**Results:**

Among the biochemical results, in AD population, malnutrition indicators (erythrocyte, hemoglobin, serum albumin, and total protein) were most significantly associated with cognitive function, as was free triiodothyronine (FT3) levels which had been observed in previous study. Malnutrition and FT3 levels depend on age but not apolipoprotein E (ApoE) genotype. Meanwhile, Among the B vitamins, TDP was the most significantly associated with malnutrition indicators and FT3.

**Conclusion:**

Our results indicated that TDP reduction could be a modifiable risk factor for malnutrition and FT3 that contributed to cognitive decline in AD patients. Correcting thiamine metabolism could serve as an optional therapy target for AD treatment.

## Introduction

Alzheimer's disease (AD) is a devastating neurodegenerative disease clinically characterized by cognitive impairment and eventual loss of self-care ability. AD is costly to treat and places a heavy burden on society and families (1). There is also a lack of effective treatment that halt or reverse disease progression. Despite considerable research have been conducted, improved understanding of AD pathogenesis and therapeutic options are desperately needed (2).

Age and ApoE genotypes are crucial for cognitive impairment. Besides, peripheral system dysfunction (3–6) and malnutrition (7, 8) have been recognized as risk factors for cognitive impairment and loss of self-care ability in AD patients. Renal (3), thyroid (4), and liver dysfunction (5) and inflammation (9) played various roles in cognitive impairment in both healthy older people and AD patients. Metabolism stress indicated by abnormal blood lipids and glucose is also considered as a potential risk for AD progression (10). Several studies reported that anemia/abnormal hemoglobin and low serum albumin level is an independent risk factor of poor cognitive performance in the elderly and for dementia and rapid cognitive decline among the elderly (11, 12). These blood biochemical results contributing to cognitive decline were regular measured and can be collected in hospital daily. However, it has not been determined which blood biochemical indicators specific are most closely associated to cognitive decline in subjects with AD.

B vitamins deficiency—especially thiamine, folate, or B12 reduction—is a leading cause of neurological impairment and disabilities of daily living (13). Populations at risk of B vitamins deficiency including the elderly, alcoholics, obesity surgery recipients, vegetarians/vegans and AD, whom are recognized as being at high risk for cognitive impairment. Several underlying pathways were indicated to link low B vitamins status and cognitive impairment as well as neuropathological changes of AD. Folate and vitamin B12 deficiency disrupted one-carbon metabolism could lead to elevate homocysteine through lowing enzymatic activities for the remethylating or trans-sulfuration (14). Alternatively which is essential for of S-adenosyl-methionine (SAM) synthesis, and required for methylation of DNA/RNA and proteins (15), its impairment caused hypermethylated redox-related genes (NUDT15 and TXNRD1). Independently, B vitamins deficiency also induced cerebrovascular dysfunction, activation of tau kinases and enhanced Aβ metabolism contributed to AD development (16–18). David Smith and colleagues' studies also indicated a possible pathogenetic role of B vitamins (B12, Folate) in mild cognitive impairment, as B vitamins therapy could abrogate effects of iron-associated brain atrophy rate (19). Thiamine diphosphate (TDP) is the bioactive form of thiamine, which acts in the production of energy from carbohydrates in the cells and is essential in the proper functioning of the nervous system (20). As a critical cofactor of three glucose metabolism enzymes (Transketolase, Pyruvate dehyrogenase complex, α-Ketoglutarate dehydrogenase complex), TDP plays important and sometimes rate-limiting roles in the brain glucose metabolism (21), TDP reduction is closely related to glucose hypometabolism and induced oxidative stress contributed to cognitive dysfunction and AD pathologies (22, 23).

Understanding the complex relationships between the different biochemical systems regulated in the peripheral system by these vitamins may facilitate AD diagnosis and improve treatment. In AD patients, the association between B vitamins status and brain function was already observed, but whether B vitamins status linked cognitive dysfunction of AD through peripheral system biochemical risk indicators remain to be clarified.

As a continuation of our previous research (24), in this study, we performed in AD population to find out some specific biochemical indicators of peripheral blood which may aggravate cognitive decline mostly. It points out that TDP related malnutrition indicators (erythrocyte, hemoglobin, serum albumin, and total protein) and FT3 are the most significant risk factors for cognitive decline in AD patients rather than other blood biochemical indicators.

## Materials and Methods

### Study Design and Subjects

From January 1, 2012 to November 30, 2020, 206 AD patients were recruited from outpatient or inpatient department of six hospitals in China (Zhongshan Hospital, Huashan Hospital, Shanghai Mental Health Center, Haiwan Hospital, Tianjin Huanhu Hospital, and Brain Hospital affiliated to Nanjing Medical University) who had no specific family history of AD. Informed consent was obtained from all participating subjects or healthcare givers. This study was approved by the Committee on Medical Ethics of hospitals.

All subjects underwent neurological examinations and neuropsychological evaluations including the Mini-Mental State Examination (MMSE), Clinical Dementia Rating (CDR), and Activity of Daily Living (ADL) scales. Their healthcare givers were also asked about the patients' abilities. All patients had cranial magnetic resonance imaging (MRI) scans within 2 weeks after questionnaires collected and were diagnosed with AD by neurologists or psychiatrists specialized in dementia (Chunjiu Zhong, Guoqiang Fei, Lei Zhao, Xiaoli Pan) according to DSM-IV criteria.

The criteria of AD patients enrollment were following: (1) primary complaint of memory decline characterized by gradual onset and continuing deterioration over 1 year; (2) MMSE score ≤ 26 (range 0–30) and at least one other cognitive deficit beyond memory impairment; (3) CDR score ≥0.5 (range 0–3) and impairment in performance of daily activities; (4) no diagnostic evidence of other neurological and mental disorders; and (5) cranial MRI excluded brain tumor, infarct, hematoma, and hydrocephalus. The exclusion criteria were: (1) major gastrointestinal tract disorders, (2) taking supplements containing B vitamins in the previous month, and (3) chronic alcohol abuse.

### Measurement of Biochemical Factors

Blood samples were collected on the same day when questionnaires completed. Blood biochemistry factors were measured using routine laboratory tests in respective hospital, including routine blood examination; glucose, lipid, and protein levels; renal, thyroid, liver function; and folate and vitamin B12 levels. Lipid metabolism was determined by levels of triglycerides, total cholesterol, and low-density lipoprotein (LDL). Thyroid function was assessed by measuring thyroid-stimulating hormone (TSH), free triiodothyronine (FT3), and free thyroxine (FT4). We selected alanine aminotransferase (ALT) and aspartate transaminase (AST) as liver function indicators, and urea, creatinine, and uric acid as the renal function indicators.

As the biologically active form of thiamine, TDP is abundant in erythrocytes and is a reliable reflection of the thiamine body store (25). Blood TDP levels were measured centralizedly in a single center within 21 days using HPLC fluoroscopy as previously described (26). The HPLC operators were blinded to participant information. Briefly, fresh samples of whole blood were immediately deproteinized with 7.4% perchloric acid. After centrifugation at 9,300 × g and 4°C for 6 min, the supernatant was collected and stored at −20°C. Thiamine phosphate esters were derivatized into thiochromes using potassium ferricyanide and separated by gradient elusion with a C18 reversed-phase analytical column (250 × 4.6 mm). The derivatives were measured by HPLC fluoroscopy on an Agilent 1100 platform (Agilent Technologies, Santa Clara, CA, USA) with excitation and emission wavelengths of 367 and 435 nm, respectively.

### ApoE Genotypes Analysis

A single-nucleotide polymorphism genotyping assay was used for the detection of ApoE alleles. Human genomic DNA was extracted and purified using a genome extraction kit (Tiangen Biotech, Beijing, China). We categorized all participants into two subgroups according to their ApoE ε4 status: an ApoE ε4 group, including the ε2/ε4, ε3/ε4, or ε4/ε4 genotypes, and a non-ApoE ε4 group, including the ε2/ε2, ε2/ε3, or ε3/ε3 genotypes.

### Statistical Analysis

Statistical analyses were performed using Statistical Package for the Social Sciences software version 18.0 (SPSS Inc., Chicago, IL, USA). Pearson's rank correlation analysis was performed to assess relationships between B vitamins and biochemical indicators. *T*-test was used to compare the difference between ApoE ε4 and non-ApoE ε4 groups. Differences were considered statistically significant as ^*^P < 0.05, ^**^P < 0.01, ^***^P < 0.001.

## Results

### Malnutrition Indicators and FT3 Levels Were Most Closely Associated With AD Cognitive Decline

To assess the relationship between biochemical parameters [organ function, lipid and glucose metabolism, inflammation cells and malnutrition (erythrocyte, hemoglobin, serum albumin and total protein)] and AD, we analyzed associations between cognitive-related scores (MMSE, ADL and CDR) and biochemical indicators in 206 sporadic AD patients aged from 48 to 101 years old in China (Table 1).

**Table 1:** Baseline characteristics.

	**All patients**	
Male/female, *n* (%)	85/121 (41/59)
Age, y	74.90 (0.73)
BMI (kg/m^2^)	21.98 (0.29)
Education, y	7.23 (0.39)
MMSE	12.34 (0.56)
ADL	16.96 (0.65)
CDR	1.72 (0.07)
**Nutrition factors**	
Erythrocyte (10^12^/L)	4.29 (0.04)
Hemoglobin (g/L)	130.2 (1.09)
Albumin (g/L)	40.26 (0.39)
Total protein (g/L)	68.86 (0.51)
**Liver function**	
AST (U/L)	22.39 (0.71)
ALT (U/L)	17.78 (0.77)
**Lipid metabolism**	
Triglyceride (mmol/L)	1.33 (0.04)
Cholesterol (mmol/L)	4.82 (0.09)
LDL (mmol/L)	2.76 (0.09)
**Glucose metabolism**	
Glucose (mmol/L)	5.68 (0.11)
**Thyroid function**	
FT3 (pmol/L)	4.06 (0.05)
FT4 (pmol/L)	15.7 (0.43)
TSH (mIU/L)	1.97 (0.09)
**Renal function**	
Urea (mmol/L)	5.73 (0.15)
Creatinine (μmol/L)	73.99 (1.82)
Uric acid (mmol/L)	294.7 (6.98)
**Inflammation**	
Leukocyte (10^9^/L)	7.53 (1.05)
Neutrophilic granulocyte (%)	63.19 (0.73)
Thrombocyte (10^9^/L)	215.80 (4.99)

*Data are presented as frequency (%) for categorical and mean (SEM) for continuous variables, unless indicated otherwise.*

*AD, Alzheimer's disease; ADL, activities of daily living; ApoE, apolipoprotein E; BMI, body mass index; CDR, Clinical Dementia Rating; MMSE, Mini Mental State Examination. ALT, alanine aminotransferase; AST, aspartate aminotransferase; FT3, free triiodothyronine; FT4, free thyroxine; LDL, low-density lipoprotein; ns, not significant; TSH, thyroid-stimulating hormone*.

Notably, the malnutrition indicators of erythrocyte, hemoglobin, albumin, and total protein levels were consistently and significantly associated with MMSE, ADL and CDR scores, with all levels of *P* < 0.001. In details, the *r* between erythrocyte, hemoglobin, albumin, and total protein levels and MMSE was 0.37, 0.37, 0.44, 0.27, respectively. As for ADL, the *r* was −0.35, −0.34, −0.43, −0.27, respectively. As for CDR, the *r* was −0.33, −0.35, −0.46, −0.30, respectively. Consistent with a previous study (6), we also observed that serum FT3 was most significantly associated with MMSE (*r* = 0.40, *P* < 0.001), ADL (*r* = −0.36, *P* < 0.001), and CDR (*r* = −0.37, *P* < 0.001) scores, while TSH was less strongly but still significantly associated with MMSE (*r* = 0.14, *P* < 0.05) and CDR (*r* = −0.20, *P* < 0.01) scores. There was no significant association between serum FT4 and cognitive function (MMSE: *r* = −0.03; ADL: *r* = 0.11; CDR: *r* = 0.04. *P* > 0.05). Besides, the liver function indicator ALT, cholesterol, glucose, leukocyte, and neutrophilic granulocyte percent mildly associated with AD cognitive scores. Other indicators were not statistically associated with AD cognitive scores (all *P* > 0.05) (Table 2).

**Table 2:** Association between biochemical indicators and cognitive dysfunction in AD patients.

**Blood biochemical markers**		**Correlation with MMSE**			**Correlation with ADL**			**Correlation with CDR**		
		***r***	***P***	***n***	***r***	***P***	***n***	***r***	***P***	***n***
Malnutrition	Erythrocyte	0.37	***	188	−0.35	***	162	−0.33	***	187
	Hemoglobin	0.37	***	201	−0.34	***	175	−0.35	***	200
	Albumin	0.44	***	169	−0.43	***	158	−0.46	***	168
	Total protein	0.27	***	167	−0.27	***	156	−0.30	***	166
Liver function	AST	−0.02	ns	178	0.04	ns	152	−0.02	ns	177
	ALT	0.16	*	197	−0.21	**	171	−0.18	*	196
Lipid metabolism	Triglyceride	0.00	ns	161	0.00	ns	135	−0.04	ns	160
	Cholesterol	0.10	ns	159	−0.24	**	133	−0.20	*	158
	LDL	0.07	ns	122	−0.15	ns	115	−0.15	ns	121
Glucose metabolism	Glucose	0.16	*	196	−0.25	**	170	−0.22	**	195
Thyroid function	FT3	0.40	***	202	−0.36	***	176	−0.37	***	201
	FT4	−0.03	ns	202	0.11	ns	176	0.04	ns	201
	TSH	0.14	*	197	−0.14	ns	172	−0.20	**	196
Renal function	Urea	−0.11	ns	189	0.09	ns	163	0.12	ns	188
	Creatinine	0.09	ns	196	−0.02	ns	170	−0.02	ns	195
	Uric acid	0.04	ns	158	0.00	ns	148	−0.04	ns	157
Inflammation	Leukocyte	0.06	ns	191	0.18	*	165	−0.06	ns	190
	Neutrophilic granulocyte %	−0.18	*	168	0.13	ns	143	0.19	*	167
	Thrombocyte	−0.06	ns	192	0.05	ns	166	0.02	ns	191

### Association Between B Vitamins Status and Blood Biochemical Indicators

To explore the potential causes of malnutrition in AD patients, we analyzed the relationship between B vitamins status and blood biochemical indicators. The results showed that blood TDP level significantly associated with erythrocyte number (*r* = 0.32, *P* < 0.001), hemoglobin level (*r* = 0.33, *P* < 0.001), albumin level (*r* = 0.30, *P* < 0.001), and weakly associated with total protein level (*r* = 0.17, *P* < 0.05). TDP levels also significantly associated with FT3 levels (*r* = 0.29, *P* < 0.001). Liver, renal function, and lipid, glucose metabolism and inflammation indicators have no statistical association with TDP level (AST: *r* = 0.09; ALT: *r* = 0.14; Urea: *r* = −0.02; Creatinine: *r* = 0.11, Uric acid: *r* = 0.10; Triglyceride: *r* = −0.04; Cholesterol: *r* = 0.00; LDL: *r* = −0.03; Glucose: *r* = 0.04; Leukocyte: *r* = −0.01; Neutrophilic granulocyte%: *r* = −0.03; Thrombocyte: *r* = 0.00, *P* > 0.05) (Table 3).

**Table 3:** Association between B vitamins status and biochemical indicators in AD patients.

**Blood biochemical markers**		**Correlation with TDP**			**Correlation with folate**			**Correlation with B12**		
		***r***	***P***	***n***	***r***	***P***	***n***	***r***	***P***	***n***
Malnutrition	Erythrocyte	0.32	***	177	0.15	*	187	0.00	ns	185
	Hemoglobin	0.33	***	190	0.13	ns	200	0.01	ns	198
	Albumin	0.30	***	162	0.19	*	168	0.05	ns	166
	Total protein	0.17	*	160	0.14	ns	166	0.04	ns	164
Liver function	AST	0.09	ns	167	−0.04	ns	177	0.07	ns	175
	ALT	0.14	ns	186	0.02	ns	196	0.19	**	194
Lipid metabolism	Triglyceride	−0.04	ns	150	0.02	ns	160	0.09	ns	158
	Cholesterol	0.00	ns	148	0.10	ns	158	0.07	ns	156
	LDL	−0.03	ns	116	0.07	ns	121	0.10	ns	119
Glucose metabolism	Glucose	0.04	ns	185	0.10	ns	195	−0.01	ns	193
Thyroid function	FT3	0.29	***	191	0.15	*	201	0.04	ns	199
	FT4	0.03	ns	191	−0.03	ns	201	0.05	ns	199
	TSH	0.01	ns	187	−0.01	ns	196	0.06	ns	194
Renal function	Urea	−0.02	ns	178	−0.06	ns	188	0.09	ns	186
	Creatinine	0.11	ns	185	−0.01	ns	195	0.07	ns	193
	Uric acid	0.10	ns	152	0.02	ns	157	0.11	ns	155
Inflammation	Leukocyte	−0.01	ns	180	0.03	ns	190	−0.02	ns	188
	Neutrophilic granulocyte %	−0.03	ns	157	−0.17	*	167	−0.14	ns	166
	Thrombocyte	0.00	ns	181	0.03	ns	191	0.06	ns	189

Meanwhile, blood folate levels weakly associated with erythrocyte number (*r* = 0.15, *P* < 0.05) and albumin level (*r* = 0.19, *P* < 0.05) but not hemoglobin or total protein level (*r* = 0.13 and *r* = 0.14, respectively, *P* > 0.05), which was similar to FT3 (*r* = 0.15, *P* < 0.05) and neutrophilic granulocyte percent (*r* = −0.17, *P* < 0.05). There were no statistical associations between folate levels and other indicators (*P* > 0.05) (Table 3). B12 level only mildly associated with liver function as reflected by ALT level (*r* = 0.19, *P* < 0.01).

### Malnutrition and FT3 Level Associated With Age but Not ApoE Genotypes

Aging is the greatest risk factor for AD development and progression, so we analyzed the relationship between peripheral organ dysfunction, malnutrition indicators and age. There were significantly negative associations with erythrocyte number (*r* = −0.40, *P* < 0.001), hemoglobin level (*r* = −0.35, *P* < 0.001), albumin level (*r* = −0.50, *P* < 0.001), total protein level (*r* = 0.34, *P* < 0.001), and FT3 (*r* = −0.49, *P* < 0.001, Table 4). Blood ALT (*r* = −0.28, *P* < 0.001), cholesterol (*r* = −0.32, *P* < 0.001), LDL (*r* = −0.27, *P* < 0.01), glucose (*r* = −0.17, *P* < 0.05), and urea (*r* = 0.20, *P* < 0.01) were also significantly associated with age (Table 4).

**Table 4:** Peripheral organs functions and nutrition homeostasis correlated with age.

**Blood biochemical markers**		**Correlation with ages**		
		***r***	***P***	***n***
Malnutrition	Erythrocyte	−0.40	***	188
	Hemoglobin	−0.35	***	201
	Albumin	−0.50	***	169
	Total protein	−0.34	***	167
Liver function	AST	0.02	ns	178
	ALT	−0.28	***	197
Lipid metabolism	Triglyceride	0.10	ns	161
	Cholesterol	−0.32	***	159
	LDL	−0.27	**	122
Glucose metabolism	Glucose	−0.17	*	196
Thyroid function	FT3	−0.49	***	202
	FT4	−0.03	ns	202
	TSH	0.05	ns	197
Renal function	Urea	0.20	**	189
	Creatinine	0.12	ns	196
	Uric acid	0.13	ns	158
Inflammation	Leukocyte	0.03	ns	191
	Neutrophilic granulocyte%	0.12	ns	168
	Thrombocyte	0.05	ns	192

**Figure 1:**
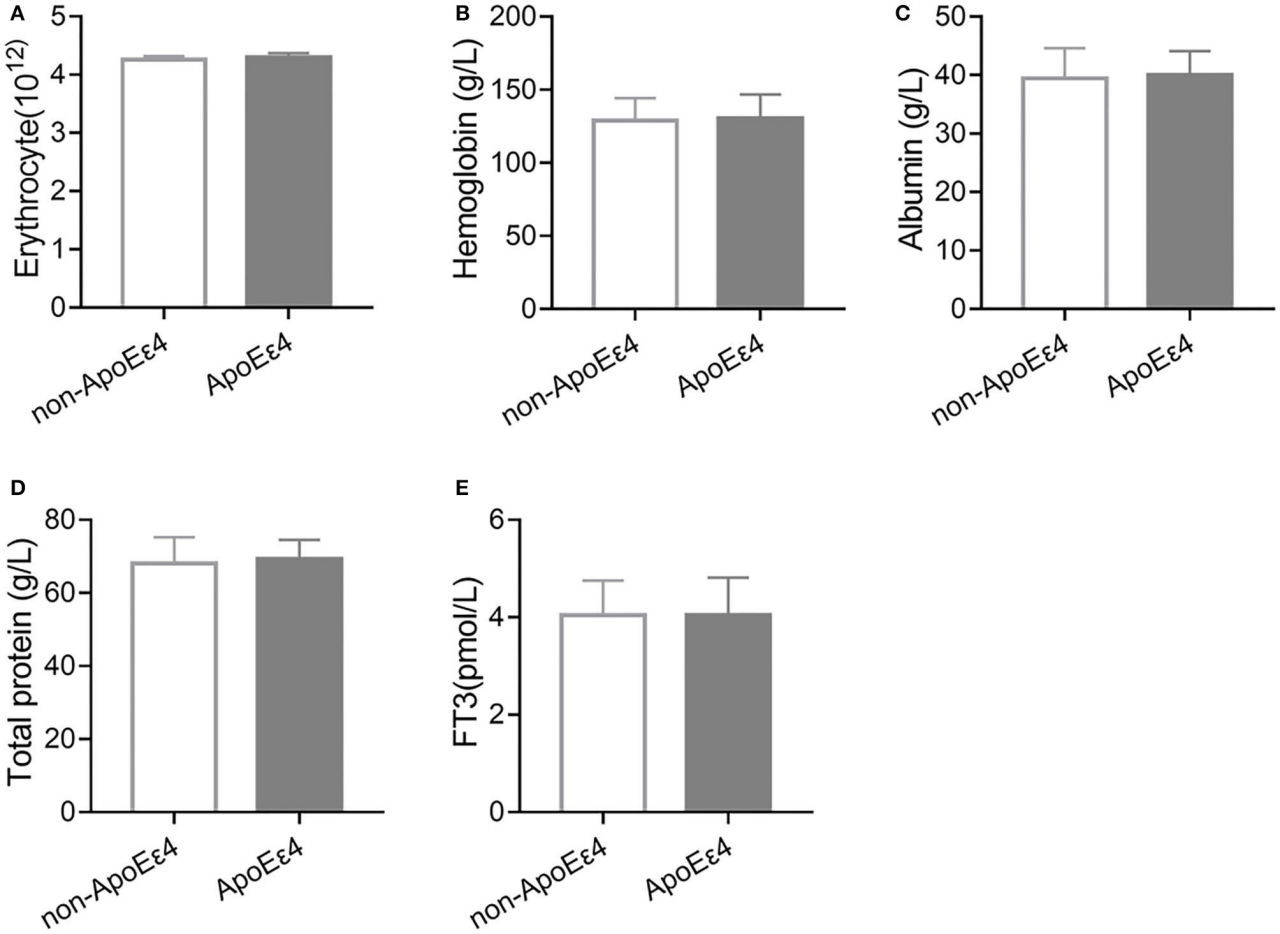
Malnutrition indicators and FT3 levels were independent of ApoE genotype. **(A)** There was no significant change in erythrocyte number between non-ApoE ε4 (*n* = 91) and ApoE ε4 (*n* = 81, *P* > 0.05) carriers. **(B)** Hemoglobin level was similar between non-ApoE ε4 (*n* = 96) and ApoE ε4 (*n* = 90, *P* > 0.05) carriers. **(C)** There was no significant difference in albumin levels between non-ApoE ε4 (*n* = 80) and ApoE ε4 (*n* = 75, *P* > 0.05) carriers. **(D)** There was no significant difference in total protein levels between non-ApoE ε4 (*n* = 79) and ApoE ε4 (*n* = 74, *P* > 0.05) carriers. **(E)** There was no significant difference in FT3 levels between non-ApoE ε4 (*n* = 95) and ApoE ε4 (*n* = 90, *P* > 0.05) carriers.

ApoE ε4 genotype is another important risk factor for AD. We detected the ApoE genotypes in a subset (86%) of AD patients in our cohort. We found no significant differences in malnutrition indicators (erythrocyte, hemoglobin, albumin, and total protein) and FT3 between non-ApoE ε4 and ApoE ε4 carrier group (Figure 1). Collectively, these results suggest that TDP related malnutrition and abnormal FT3 level were dependent on age but not ApoE genotypes.

## Discussion

In this cross-sectional study, we firstly investigated most blood biomarkers to assess the association between peripheral systemic function and cognition scores in AD population. We found that malnutrition indicators consisted of erythrocyte, hemoglobin, serum albumin and total protein associated with AD cognitive scores most, as well as FT3, which is consistent with previous study (6). Among B vitamins, TDP was most significantly associated with malnutrition indicators and FT3. Malnutrition indicators and FT3 status associated with age but not ApoE genotypes.

It is widely accepted that AD is a neurodegenerative disease that involves multiple organ systems. Here we found that peripheral systemic dysfunction—especially malnutrition and FT3—significantly associated with cognitive decline in AD patients. A previous study reported that a poorer Mini-Nutritional Assessment score, lower body mass index, lower fat-free mass, and a less healthy dietary pattern were associated with a higher risk of 2-year clinical progression of AD (8). Serum FT3 levels were associated with the risk of progression to AD (6). These two risk factors both associated with TDP level significantly, indicating that they may interact with each other through some common pathways to influence the development of AD. Understand the potential mechanism may promote the diagnosis and assess the prognosis of the subsequent cognitive decline in AD.

To our knowledge, others consistently showed that low blood hemoglobin level was associated with AD cognitive impairment (12, 18). Anemia is commonly present as thiamine deficiency (27). Gene mutations related to thiamine metabolism are often found in patients with thiamine-responsive megaloblastic anemia (27). A large population study showed that a micronutrient-fortified rice diet thiamine and other nutrients significantly improved hemoglobin levels, anemia prevalence, and cognitive performance among schoolchildren in India (28). At the molecular level, precursor porphyrins are the major component of hemoglobin that are synthesized in the mitochondria using the production of succinyl-coenzyme A (CoA) from the Krebs cycle (29). However, the mitochondrial pool of succinyl-CoA may limit heme biosynthesis in the setting of TDP deficiency. Decreased heme biosynthesis in erythroid cells then causes anemia (30). Previous studies also showed that thiamine deficiency induced anorexia by inhibiting the hypothalamic adenosine monophosphate-activated protein kinase signal pathway and disrupting neuroendocrine feedback control of food intake and energy metabolism, which may be related to malnutrition in AD patients (31). Although adults with vitamin B12 or folate deficiency can also present with megaloblastic anemias, our results suggest that anemia in AD patients may be caused by TDP reduction more than folate or B12 deficiency. Previous studies showed that low albumin was an independent risk marker for cognitive decline in older adults (32). Experimental and clinical studies suggested that the disturbed metabolism of thiamine could lead to tissue-specific thiamine deficiency in the kidney and increase albumin excretion and microalbuminuria in peripheral nephropathy (33). This probably occurs by impairing the activity and expression of TDP-dependent enzymes—particularly transketolase, and relating to increase of low-grade vascular inflammation and impairment of glomerular and tubular structure (34, 35). It maybe the potential pathological process for low levels of albumin or/and total protein in AD. We also observed that FT3 associated with progression of cognitive decline in AD as before (6). A possible association is that the conversion of FT3 to the acetate would proceed by the thiamine-requiring pyruvic dehydrogenase system. Thus, a thiamine deficiency could block thyroid function and consequently reduce oxidative metabolism and energy production by reducing the formation of FT3 (36). Others and our studies have reported that supplement of benfotiamine (as a synthetic thiamine precursor) is efficacious in improving cognitive function in AD patients (37, 38). While Aisen et al. found that supplement of high-dose B vitamins (folate, vitamin B6 and B12) had no beneficial effect on the primary cognitive measure in individuals with mild to moderate AD, although total homocysteine declined (39). As a continuous study, our results also suggested that proper TDP level may play an outstanding role in the maintenance of nutrition homeostasis and cognitive function in AD patients. However, the links of thiamine deficiency with malnutrition in AD were not well-investigated.

TDP reduction was an AD-specific pathological feature that closely correlated with brain glucose metabolism (22). Many researches have demonstrated the association between the thiamine deficiency and cognition impairment in animals. Thiamine deficiency resulted in reduced activity of the crucial acetylcholine synthetic enzyme, choline acetyltransferase and neurogenesis (20). Besides, thiamine deficiency could induce excess glutamate release and selective sub-medial thalamic nucleus death involving both inflammation and oxidative stress in the brain (20). Our results indicated that low TDP may be a link between broken nutrition homeostasis in the peripheral system and brain glucose hypometabolism in AD patients. Thiamine uptake is enhanced by thiamine deficiency and reduced by thyroid hormone and diabetes (40). However, how thiamine or its derivatives protect against malnutrition and abnormal FT3 levels in older subjects or AD patients requires further investigation. According to the “homocysteine hypothesis”, folate and vitamins B6 and/or B12 deficiency could contribute to cognitive decline, Aβ deposition, neurofibrillary tangles, white matter damage and brain atrophy through DNA methylation, histone modifications and microRNAs (41–44), while the researches relating epigenetics on TDP pathology in AD are still limited and need to be further explored. Although how the malnutrition and TDP reduction work in cognitive decline are still ambiguous and more efforts are needed to further explore, it is positive that correcting thiamine metabolism could be a more effective treatment strategy in patients with AD (45).

In conclusion, malnutrition and FT3 are important modifiable factors for AD progression. Thiamine has been known for protecting against the neuropathy, and TDP may be a link to explore the association among different organs metabolism function, further a potential target to understand the mechanism of cognitive decline. B vitamins supplementation, especially thiamine or its analogs, may be beneficial to prevent cognitive decline.

## Data Availability Statement

The raw data supporting the conclusions of this article will be made available by the authors, without undue reservation.

## Ethics Statement

The studies involving human participants were reviewed and approved by Committee on Medical Ethics of Zhongshan Hospital, Committee on Medical Ethics of Huashan Hospital, Committee on Medical Ethics of Shanghai Mental Health Center, Committee on Medical Ethics of Haiwan Hospital, Committee on Medical Ethics of Tianjin Huanhu Hospital, and Committee on Medical Ethics of Brain Hospital affiliated to Nanjing Medical University. The patients/participants provided their written informed consent to participate in this study.

## Author Contributions

XC, LZ, GF, and CZ conceived the study designed experiments. TQ, XP, SS, CW, and GF performed patients collection, thiamine metabolite detection, and ApoE genotype analysis. TQ, LZ, and XC wrote the paper. All authors read and approved the final version of the manuscript.

## Funding

This study was supported by grants from the National Natural Science Foundation of China (81901081, 81870822, 91332201, 82171408, 8217051360, and 8150050116), Shanghai Municipal Science and Technology Major Project (No. 2018SHZDZX01) and ZJ Lab, Science and Technology Commission of Shanghai Municipality (20ZR1411300), and the Natural Science Foundation of Fujian Province (2020CXB049).

## Conflict of Interest

The authors declare that the research was conducted in the absence of any commercial or financial relationships that could be construed as a potential conflict of interest.

## Publisher's Note

All claims expressed in this article are solely those of the authors and do not necessarily represent those of their affiliated organizations, or those of the publisher, the editors and the reviewers. Any product that may be evaluated in this article, or claim that may be made by its manufacturer, is not guaranteed or endorsed by the publisher.
